# Tumor Cells Positive and Negative for the Common Cancer Stem Cell Markers Are Capable of Initiating Tumor Growth and Generating Both Progenies

**DOI:** 10.1371/journal.pone.0054579

**Published:** 2013-01-21

**Authors:** Sheng-Dong Huang, Yang Yuan, Hao Tang, Xiao-Hong Liu, Chuan-Gang Fu, He-Zhong Cheng, Jian-Wei Bi, Yong-Wei Yu, De-Jun Gong, Wei Zhang, Jie Chen, Zhi-Yun Xu

**Affiliations:** 1 Institute of Cardiothoracic Surgery, Changhai Hospital, Second Military Medical University, Shanghai, P. R. China; 2 Department of Colorectal Surgery, Changhai Hospital, Second Military Medical University, Shanghai, P. R. China; 3 Department of General Surgery, Changhai Hospital, Second Military Medical University, Shanghai, P. R. China; 4 Department of Pathology, Changhai Hospital, Second Military Medical University, Shanghai, P. R. China; 5 Department of Hematology, Changhai Hospital, Second Military Medical University, Shanghai, P. R. China; 6 Department of Cardiothoracic Surgery, Changhai Hospital, Second Military Medical University, Shanghai, P. R. China; Southern Illinois University School of Medicine, United States of America

## Abstract

The cancer stem cell (CSC) model depicts that tumors are hierarchically organized and maintained by CSCs lying at the apex. CSCs have been “identified” in a variety of tumors through the tumor-forming assay, in which tumor cells distinguished by a certain cell surface marker (known as a CSC marker) were separately transplanted into immunodeficient mice. In such assays, tumor cells positive but not negative for the CSC marker (hereby defined as CSC^+^ and CSC^−^ cells, respectively) have the ability of tumor-forming and generating both progenies. However, here we show that CSC^+^ and CSC^−^ cells exhibit similar proliferation in the native states. Using a cell tracing method, we demonstrate that CSC^−^ cells exhibit similar tumorigenesis and proliferation as CSC^+^ cells when they were co-transplanted into immunodeficient mice. Through serial single-cell derived subline construction, we further demonstrated that CSC^+^ and CSC^−^ cells from CSC marker expressing tumors could invariably generate both progenies, and their characteristics are maintained among different generations irrespective of the origins (CSC^+^-derived or CSC^−^-derived). These findings demonstrate that tumorigenic cells cannot be distinguished by common CSC markers alone and we propose that cautions should be taken when using these markers independently to identify cancer stem cells due to the phenotypic plasticity of tumor cells.

## Introduction

A fundamental question in the field of tumor research is which cells can initiate tumors. Two models have been put forward to explain the initiation of tumors [1], [2]. The clonal evolution model (also known as the stochastic model) implies that tumors comprise cells with equal tumorigenic potential and that any functional heterogeneity is attributable to random or stochastic influences (intrinsic or extrinsic) that may alter the behavior of individual cells in the tumor. By contrast, the cancer stem cell (CSC) model (also known as the hierarchy model) argues that, like normal tissues, which are cellular hierarchies maintained by stem cells, tumors can be explained by hierarchical organizations, in which CSCs lying at the apex hold the capacity for tumor initiation, self-renewal, and generation of phenotypically diverse cells with no or limited proliferative capacity. Advocates of the CSC model propose that CSCs may account for tumor behaviors such as metastasis [3], [4] and resistance to chemotherapy or radiotherapy [5]–[9]. Hence, CSC-targeted therapy may be the future direction of tumor treatment [10]–[13].

Through tumor-forming assay in which phenotypically diverse cells were separately transplanted into immunodeficient mice, CSC was first “identified” in human acute myeloid leukemia (AML) since only CD34^+^CD38^−^ cells were found to have the ability of tumor initiation, self-renewal, and generating cells of other subsets under such condition [14]. Since then, the xenotransplantation experimental model has been widely used in CSC studies. Using various cell surface markers, a large body of literature has been published suggesting the existence of CSCs in a variety of tumors such as chronic myeloid leukemia (CML) [15], [16], acute promyelocytic leukemia (APL) [17], [18], breast cancer [19], glioblastoma [20]–[23], colon cancer [24]–[26] and melanoma [27]–[30].

However, there is unsettled controversy as to whether the tumor-forming capacity of human tumor cells was correctly reflected in previous studies [31], [32]. Since the efficiency of xenotransplantation in the majority of cases is considerably lower than that for syngeneic transplants, Kelly et al. suggested that the tumor-forming capacity of human tumor cells might be seriously compromised in the mouse milieu due to species-specific differences in the affinity (or recognition) of cytokine and growth factor receptors for their cognate ligands [33]. Besides, Quintana et al. employed a more highly immunocompromised mouse strain (NOD/SCID interleukin-2 receptor gamma chain null [ll2rg−/−]) for xenotransplantation assay and found that this could dramatically increase the detectable frequency of cells with tumorigenic potential in human melanoma, suggesting that the tumor-forming capacity of human tumor cells could be greatly compromised due to immune influence in the foreign milieu [34]. These led us to question whether the proliferative and tumorigenic capacity of human tumor cells, especially that of the “non-CSCs” could have been underestimated in the previous studies.

In the present study, we evaluated the proliferation and apoptosis of the putative CSCs (CSC^+^ cells) and non-CSCs (CSC^−^ cells) in primary tumors as well as tumor cell lines by flow cytometry. In contrast to the previous reports from conventional xenotransplantation assays (transplanting CSC^+^ and CSC^−^ cells separately), where CSC^−^ cells were shown to have no or limited proliferative capacity [1], [35], [36], we found no significant differences in proliferation and apoptosis between the two subsets in the native state. We further employed a cell tracing technique to follow the proliferation, tumorigenicity of CSC^+^ and CSC^−^ cells. We chose to study cells from several tumor cell lines instead of primary tumors, which might comprise genetically diverse cells [27], [37]. It was found that CSC^−^ cells exhibited similar proliferative and tumorigenic capacities as CSC^+^ cells when the two subsets coexisted. Moreover, both subsets could give rise to CSC^+^ as well as CSC^−^ progenies, while the characteristics of the CSC^+^ (or CSC^−^) cells were maintained among different generations regardless of their origins (CSC^+^-derived or CSC^−^-derived). Our results suggest that the CSC markers should be used judiciously to differentiate cancer stem cells from other tumor cells due to their phenotypic plasticity.

## Results

### Expression of CSC markers varies enormously in human primary tumors and tumor cell lines

**Figure 1:**
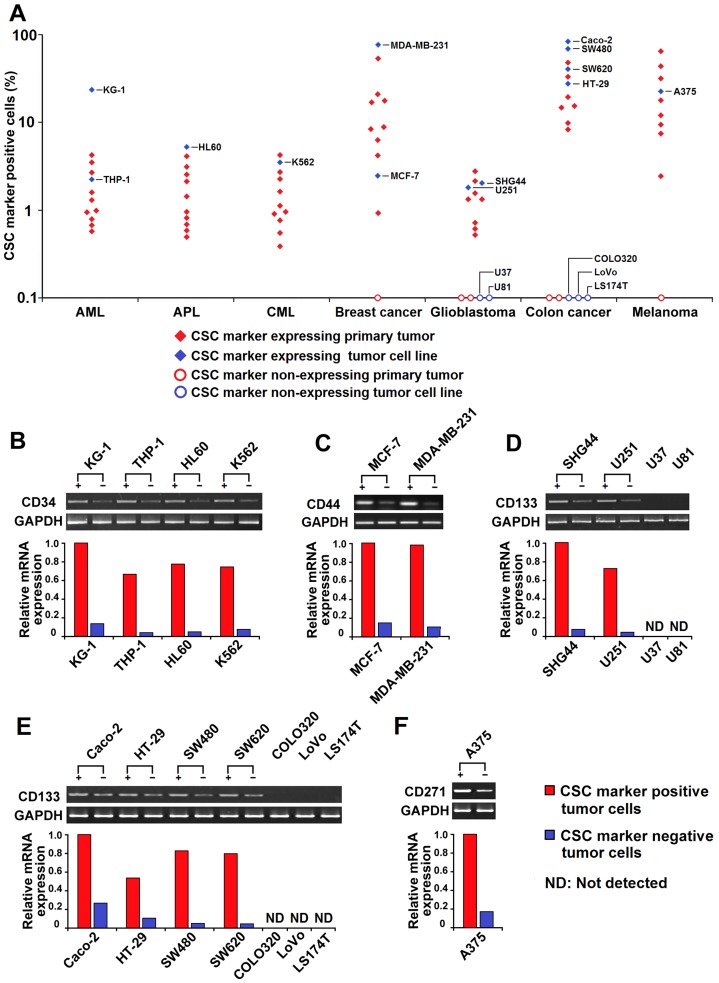
Expression of CSC marker in human primary tumors and tumor cell lines. (**A**) The percentage of CSC marker positive cells from human primary tumors (n = 10 for each type) and tumor cell lines. Single cell suspensions were prepared, followed by flow cytometric analysis. Dots represent the percentage of CD34^+^CD38^−^ cells in AML, APL, and CML, CD44^+^CD24^−^ cells in breast cancer, CD133^+^ cells in glioblastoma and colon cancer, and CD271^+^ cells in melanoma samples. (**B–F**) The CSC marker mRNA expression level of cells (CSC^+^ and CSC^−^ cells from CSC marker expressing tumor cell lines, and cells from non-expression tumor cell lines) was analyzed by qRT-PCR and normalized to *GAPDH*. The level of CD34 mRNA in KG-1 CSC^+^ cells, CD44 mRNA in MCF-7 CSC^+^ cells, CD133 mRNA in SHG-44 and Caco-2 CSC^+^ cells, CD271 mRNA in A375 CSC^+^ cells, was arbitrarily designated as 1.0 for leukemia, breast cancer, glioblastoma, colon cancer, melanoma samples, respectively. Photographs show the RT-PCR products and histograms show the CSC marker mRNA expression level of cells from leukemia (**B**), breast cancer (**C**), glioblastoma (**D**), colon cancer (**E**), and melanoma (**F**) cell lines. Note that cells from CSC marker non-expressing tumor cell lines (U37, U81, COLO320, LoVo, LS174T) do not express CSC marker mRNA.

Previous studies suggested that leukemogenic/tumorigenic cells were restricted to a rare population of tumor cells expressing certain CSC markers [35], [36]. Using a trypsin-free dissociation protocol (to maintain epitope integrity), we detected the percentage of CSC marker positive cells (e.g., CD34^+^CD38^−^ for AML, APL, and CML, CD44^+^CD24^−^ for breast cancer, CD133^+^ for glioblastoma and colon cancer, and CD271^+^ for melanoma) in human primary tumors as well as tumor cell lines by flow cytometric analysis. We found that a considerable number of primary tumors or tumor cell lines do not express those CSC markers (Figure 1A), which is consistent with some other reports [38], [39]. In addition, we performed quantitative RT-PCR and found that cells (both CSC^+^ and CSC^−^) from CSC marker expressing tumors, but not cells from CSC marker non-expressing tumors, express CSC marker mRNA (Figures 1B–F and Figure S1), suggesting that the CSC marker expressing and non-expressing tumors might originate from different types of tumor cells. In primary tumors and tumor cell lines expressing those CSC markers, the percentage of CSC marker positive cells varied enormously, ranging from 0.4% in an AML to as high as 82.7% in a colon cancer (Figure 1A).

### CSC^−^ tumor cells exhibit similar proliferation as CSC^+^ tumor cells in the native state

**Figure 2:**
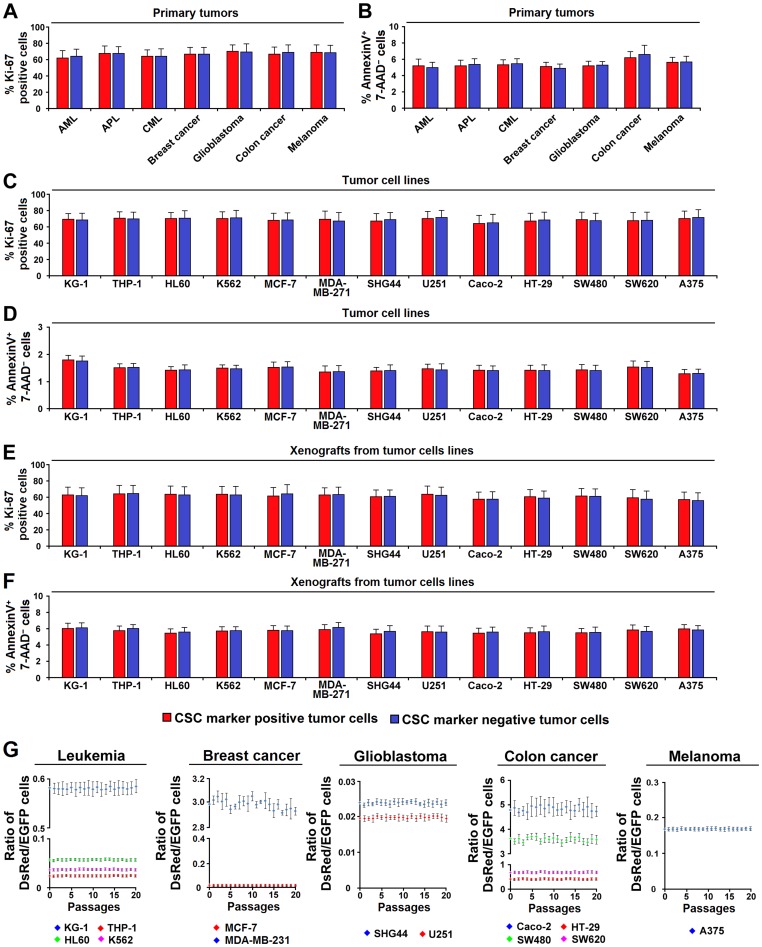
CSC^+^ and CSC^−^ tumor cells display similar proliferative capacity in the native state. (**A, B**) CSC marker expressing primary sample were selected to undergo Ki-67 expression and apoptosis analysis. Data represent mean ± SEM; n = 10 for each type of leukemia, n = 8 for glioblastoma and for colon cancer, n = 9 for breast cancer and for melanoma. (**A**) Ki-67 expression of CSC^+^ and CSC^−^ cells from human primary tumors. Single cell suspensions of human primary tumors were stained with antibodies specific to the CSC markers and Ki-67-FITC, followed by flow cytometric analysis. (**B**) Apoptosis assay of CSC^+^ and CSC^−^ cells from human primary tumors. Single cell suspensions of human primary tumors were stained with antibodies specific to the CSC markers and Annexin V-FITC/7-ADD, followed by flow cytometric analysis. (**C–D**) Ki-67 expression (**C**) and apoptosis (**D**) assay of CSC^+^ and CSC^−^ cells from human tumor cell lines cultured in serum-containing medium. Data represent mean ± SEM from 3 independent experiments. (**E–F**) Ki-67 expression (**E**) and apoptosis (**F**) assay of CSC^+^ and CSC^−^ cells of xenografts derived from human tumor cell lines. Data represent mean ± SEM from 3 independent experiments. (**G**) DsRed-labeled CSC^+^ and EGFP-labeled CSC^−^ cells originating from the same tumor cell lines were mixed according to their original ratios and co-cultured in serum-containing medium for 20 passages. Dots show the ratio of CSC^+^-derived to CSC^−^-derived (DsRed:EGFP) cells at different passages. Data represent mean ± SEM from 3 independent experiments.

To investigate the proliferation of the putative CSCs (CSC^+^ cells) and non-CSCs (CSC^−^ cells) in the native state, we measured the percentage of proliferating and apoptotic cells of the two subsets in CSC marker expressing primary tumors (e.g. AML, APL, CML, breast cancer, glioblastoma, colon cancer, and melanoma) by flow cytometry. As shown in Figures 2A and 2B, the percentage of Ki-67 positive cells, and the percentage of Annexin V^+^ 7-AAD^−^ cells did not differ significantly between the two subsets. Similar results were obtained in *in vitro* (Figures 2C and 2D) and *in vivo* (Figures 2E and 2F) studies of CSC marker expressing human tumor cell lines. These results suggest that the proliferative capacity of CSC^−^ cells is similar to that of CSC^+^ cells in the native state.

To follow the proliferation of CSC^+^ and CSC^−^ cells, we further employed a cell tracing technique designed to simulate the *in situ* environment in which both subsets coexist. DsRed-labeled CSC^+^ and EGFP-labeled CSC^−^ cells originating from the same tumor cell lines were mixed according to their original ratios and co-cultured in serum-containing medium for 20 passages (see Experimental Procedures for details). Flow cytometry analysis showed that the ratio of CSC^+^-derived to CSC^−^-derived (DsRed:EGFP) cells remained basically unchanged throughout the whole process (Figure 2G). These data suggest that both CSC^+^ and CSC^−^ cells could propagate extensively.

### CSC^−^ tumor cells show similar tumor-forming capacity as CSC^+^ tumor cells upon co-transplantation

**Figure 3:**
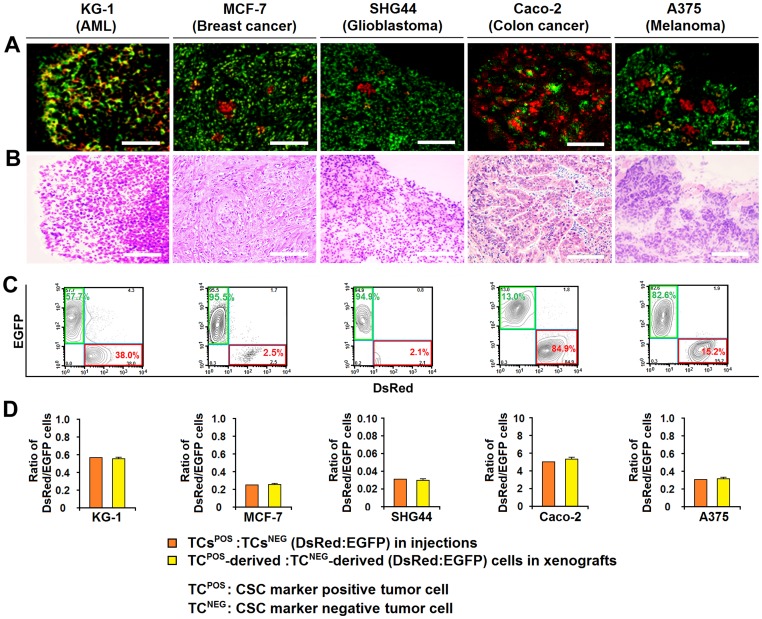
CSC^+^ and CSC^−^ tumor cells display similar tumor-forming capacity upon co-transplantation. (**A and B**) DsRed-labeled CSC^+^ and EGFP-labeled CSC^−^ cells originating from the same tumor cell lines were mixed to their original ratio and co-transplanted into sublethally irradiated NOD-SCID mice. Photographs represent fluorescent images (**A**) and hematoxylin and eosin staining (**B**) of serial sections of the xenografts derived from KG-1, MCF-7, SHG44, Caco-2 and A375. Scale bar  = 100 μm. (**C**) Flow cytometric contour plots show the percentage of DsRed and EGFP cells in the xenografts. (**D**) Histograms show the ratio of CSC^+^ to CSC^−^ (DsRed:EGFP) cells in injections and the ratio of CSC^+^-derived to CSC^−^-derived (DsRed:EGFP) cells in xenografts. Data represent mean ± SEM from 3 independent experiments.

In light of the above findings, we investigated the tumor-forming capacity of CSC^+^ and CSC^−^ cells by co-transplantation as well as conventional xenotransplantation (transplanting CSC^+^ and CSC^−^ cells separately). DsRed-labeled CSC^+^ and EGFP-labeled CSC^−^ cells originated from the same tumor cell lines were mixed in their original ratio. Different numbers of CSC^+^, CSC^−^, or mixed cells were injected under the renal capsule of sublethally irradiated NOD-SCID mice, respectively. It was found that both CSC^+^ and CSC^−^ cells could initiate tumor formation when transplanted separately, although the frequency of tumor formation by CSC^−^ cells was significantly lower than that in CSC^+^ cells (Table S1). When CSC^+^ and CSC^−^ cells were co-transplanted, the xenografts were composed of both CSC^+^-derived (DsRed) and CSC^−^-derived (EGFP) cells, as shown in Figures 3A and 3B. Importantly, flow cytometry analysis showed that the ratios of CSC^+^-derived to CSC^−^-derived (DsRed:EGFP) cells in the xenografts were not significantly different from the ratios of CSC^+^ to CSC^−^ (DsRed:EGFP) cells in the mixtures that had been injected (Figures 3C and 3D, Table 1). We carried out the xenotransplantation down to the third passage. Flow cytometry analysis showed that the ratios of CSC^+^-derived to CSC^–^-derived (DsRed:EGFP) cells in the xenografts remained basically unchanged among the different passages (Table 1). These data suggest that although CSC^+^ cells can be more tumorigenic when studied separately, pre-isolated CSC^−^ cells can be sustained in the co-transplatation model. The studies also imply that the tumor-forming capacity of CSC^−^ cells could be underestimated if they are studied separately in a foreign milieu.

**Table 1:** Tumorigenesis analysis of co-transplanted CSC^+^ and CSC^−^ tumor cells.

Cell source	CSC^+^: CSC^−^ cells (DsRed:EGFP) in injections	Xenografts/ Injections	CSC^+^-derived: CSC^−^-derived cells (DsRed:EGFP) in xenografts		
			Passage1	Passage2	Passage3
KG-1	0.572	3/3	0.563±0.010	0.585±0.012	0.569±0.009
THP-1	0.022	3/3	0.021±0.005	0.024±0.003	0.023±0.004
HL60	0.055	3/3	0.059±0.008	0.056±0.012	0.057±0.011
K562	0.036	3/3	0.034±0.007	0.037±0.009	0.037±0.006
MCF-7	0.025	3/3	0.024±0.008	0.025±0.010	0.024±0.008
MDA-MB-231	2.986	3/3	3.019±0.065	3.039±0.068	2.954±0.094
SHG44	0.022	3/3	0.025±0.006	0.024±0.005	0.023±0.005
U251	0.019	3/3	0.022±0.005	0.020±0.005	0.022±0.006
Caco-2	4.780	3/3	5.178±0.299	4.980±0.273	4.882±0.313
HT-29	0.408	3/3	0.419±0.012	0.399±0.010	0.408±0.015
SW480	3.545	3/3	3.631±0.158	3.638±0.187	3.561±0.117
SW620	0.695	3/3	0.709±0.015	0.703±0.016	0.686±0.012
A375	0.164	3/3	0.171±0.011	0.167±0.009	0.168±0.011

DsRed-labeled CSC^+^ and EGFP-labeled CSC^−^ cells originating from the same tumor cell lines were mixed according to their original ratios and co-transplanted into the mice (n = 3, 1×10^5^ cells per mouse) to generate the xenografts (arbitrarily classified as Passage1). 1×10^5^ cells isolated from Passage1 xenografts were transplanted into the mice to generate the Passage2 xenografts. Likewise, the passage3 xenografts were generated from cells of passage2 xenografts. The ratio of CSC^+^-derived to CSC^−^-derived (DsRed:EGFP) cells in the xenografts was analyzed by flow cytometry. Notably, the ratios of CSC^+^-derived to CSC^−^-derived (DsRed:EGFP) cells in the xenografts of each and every passage were not significantly different from the ratios of CSC^+^ to CSC^−^ (DsRed:EGFP) cells in the mixtures that had been injected.

### Plasticity of CSC marker based hierarchy in CSC marker expressing tumors

**Figure 4:**
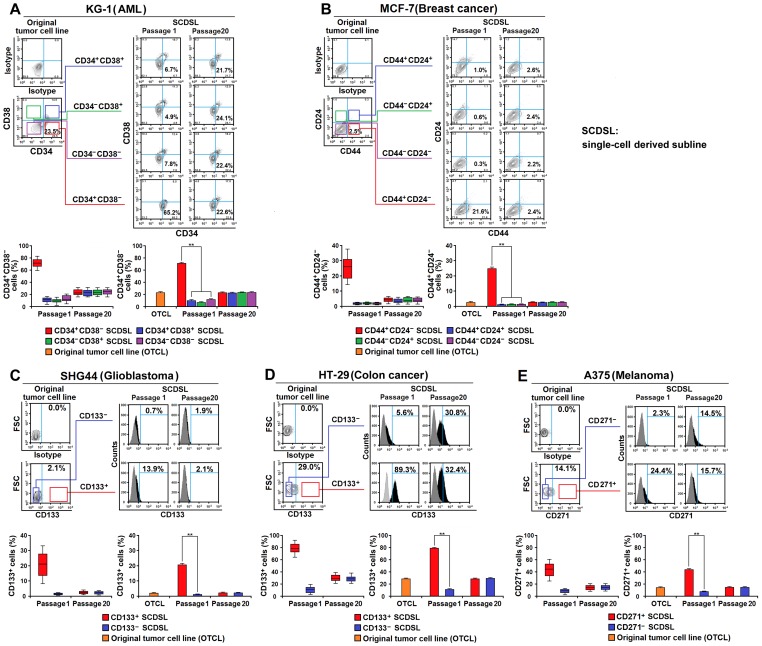
CSC^−^ and CSC^+^ tumor cells can generate both progenies. (**A–E**) CSC^+^ and CSC^−^ cells from the original tumor cell lines were selected to generate single-cell derived sublines (SCDSLs). The percentage of CSC marker positive cells in the original tumor cell lines or SCDSLs (passage 1 and passage 20) of KG-1 (**A**), MCF-7 (**B**), SHG44 (**C**), HT-29 (**D**), and A375 (**E**) was analyzed by flow cytometry. Box plots show the percentage of CSC marker positive cells in SCDSLs, with the whiskers representing the minimum and maximum values, the central lines representing the median value, and the boxes representing the 25th and 75th percentile. Histograms show the percentage of CSC marker positive cells in original tumor cell lines and SCDSLs. Data of SCDSLs represent mean ± SEM from 100 samples; Data of original tumor cell lines represent mean ± SEM from 3 independent experiments; ** *P*<0.01 by independent *t*-test.

Unexpectedly, we found that a considerable number of CSC^+^ cells were present in the CSC^−^-derived populations both *in vitro* and *in vivo* and the percentage of CSC^+^ cells in the CSC^+^- and CSC^−^-derived populations were comparable after cultured (or xenotransplanted) for several passages (Table S2), suggesting that CSC^−^ cells in CSC marker expressing tumors may give rise to CSC^+^ cells. To confirm that the CSC^+^ cells in CSC^−^-derived population were indeed progenies of CSC^−^ cells and not a result of cell contamination introduced during cell sorting [40], we measured (in CSC marker expressing tumor cell lines, using flow cytometry) the percentage of CSC marker positive cells in single CSC^+^-derived or CSC^−^-derived sublines. As shown in Figure 4 and Figure S2, both CSC^+^ and CSC^−^ cells were capable of generating single cell-derived sublines and giving rise to the two subsets. Initially, the percentage of CSC marker positive cells in single CSC^−^-derived sublines was significantly lower than in single CSC^+^-derived sublines. However, over an extended period of culture (approximately 20 passages, see Experimental Procedures for details), the percentage of CSC marker positive cells in each subline became statistically similar to that of the original tumor cell lines. In addition, we found that the clone forming efficiency of CSC^−^ cells ranged from 36.1% in Caco-2 to 70.3% in THP-1 (Figure S3). Notably, the percentage of clone forming CSC^−^ cells is far higher than the rate of contamination by CSC^+^ cells (false negative cells, usually lower than 0.1%) in the CSC^−^ population as sorted by FACS. These results indicate that CSC^−^ cells can give rise to CSC^+^ cells in CSC marker expressing human tumors.

**Figure 5:**
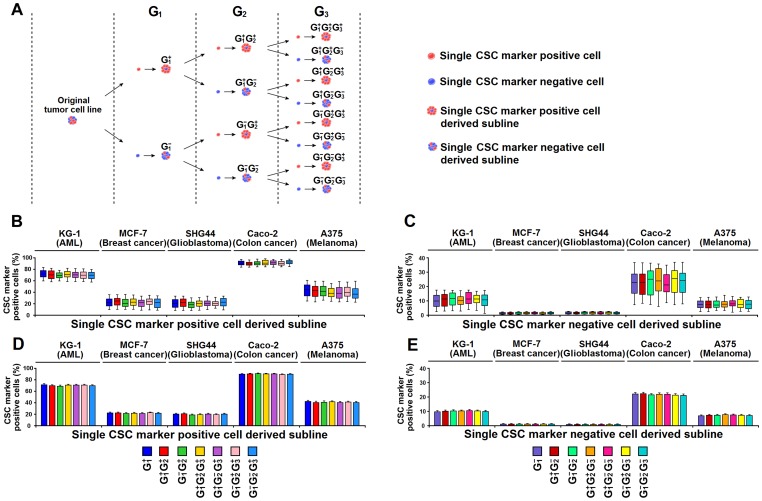
There is no CSC marker based hierarchy in CSC marker expressing tumors. (**A**) Schematic diagram shows the construction of serial single-cell derived sublines (SCDSLs). CSC^+^ and CSC^−^ cells from the original tumor cell lines were selected to generate SCDSLs and arbitrarily classified as generation 1 SCDSLs (including G_1_^+^ and G_1_^−^). CSC^+^ and CSC^−^ cells from G_1_ SCDSLs were selected to generate G_2_ SCDSLs (including G_1_^+^G_2_^+^, G_1_^−^G_2_^+^, G_1_^+^G_2_^−^ and G_1_^−^G_2_^−^). We repeated the procedure until G_3_ SCDSLs were obtained. **(B–E)** The percentage of CSC marker positive cells in SCDSLs from KG-1, MCF-7, SHG44, Caco-2 and A375 was analyzed by flow cytometry when the cell quantity reached approximately 1×10^6^. Box plots show the percentage of CSC marker positive cells in single CSC^+^-derived (**B**) and CSC^−^-derived (**C**) sublines from different generations, with the whiskers representing the minimum and maximum values, the central lines representing the median value, and the boxes representing the 25th and 75th percentile. Histograms show the percentage of CSC marker positive cells in single CSC^+^-derived (**D**) and CSC^−^-derived (**E**) sublines from different generations. Data represent mean ± SEM from 100 samples, each from one independent serial SCDSL construction.

**Figure 6:**
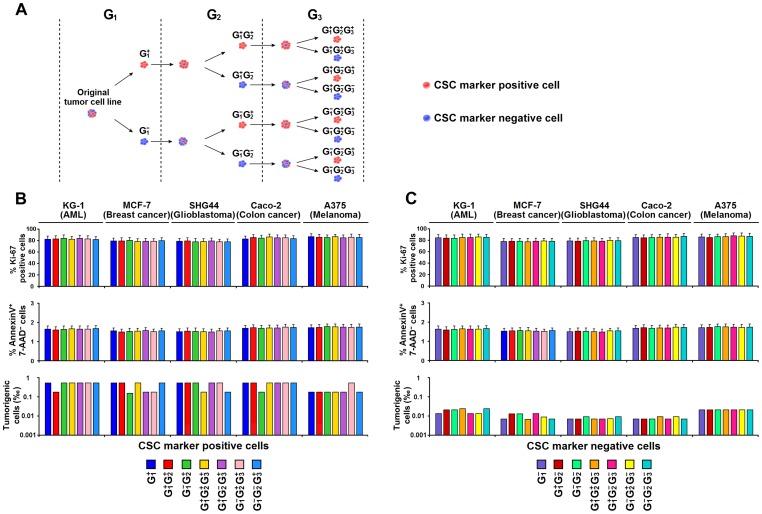
Proliferation and tumorigenesis of the CSC^+^ (or CSC^−^) cells from different generations are comparable. (**A**) Schematic diagram shows the cell classification based on generation and CSC marker expression. Cells from the original tumor cell lines were arbitrarily classified as generation 1 (G_1_) cells and sorted into CSC marker positive (G_1_^+^) and negative (G_1_^−^) cells. G_1_^+^ and G_1_^−^ cells were then propagated (from 1×10^2^ to approximately 1×10^8^) to generate G_2_ (including G_1_^+^G_2_^+^, G_1_^+^G_2_^−^, G_1_^−^G_2_^+^ and G_1_^−^G_2_^−^) cells. We repeated the procedure until G_3_ cells were obtained. (**B and C**) CSC^+^ and CSC^−^ cells from different generations were used for proliferative and tumorigenic assays. Histograms show the percentage of Ki-67 positive cells, the percentage of Annexin V^+^ 7-AAD^−^ cells, and the frequency of tumorigenic cells of CSC^+^ cells (**B**) and CSC^−^ cells (**C**) from different generations in KG-1, MCF7, SHG44, Caco-2 and A375. Frequency of tumorigenic cells was calculated using Extreme Limiting Dilution Analysis software. Other data are expressed as mean ± SEM from 3 independent experiments.

We carried the single-cell derived subline construction down to the third generation (Figure 5A). Regardless of the generations or origins (CSC^+^-derived or CSC^−^-derived), each and every subline contained a considerable number of CSC^+^ cells (Figures 5B and 5C, Figures S4A and S4B), and the percentage of CSC marker positive cells in the single CSC^+^-derived (or CSC^−^-derived) sublines was comparable among all generations (Figures 5D and 5E, Figures S4C and S4D). Specifically, both subsets could invariably generate CSC^+^ and CSC^−^ progenies in all generations, and no sublines consisted exclusively of CSC^+^ or CSC^−^ cells. Moreover, we studied the proliferation and tumorigenesis of the CSC^+^ (or CSC^−^) cells from different generations (Figure 6A). As shown in Figures 6B, 6C and Figure S5, the percentage of Ki-67 positive cells, the percentage of Annexin V^+^ 7-AAD^−^ cells, and the frequency of tumorigenic cells were statistically similar across all generations despite of the origins. These results show that CSC^−^ cells are capable of generating CSC^+^ cells and suggest that there is considerable plasticity of these common CSC markers in these tumors.

## Discussion

The question of which tumor cells contribute to tumor progression has fundamental implications for therapy. Based mainly on the findings that only tumor cells expressing certain cell surface markers could initiate tumor growth when transplanted into immunodeficient mice, advocates of the CSC model propose that tumors are hierarchically organized and hence tumor therapy should be directed at eliminating the tumorigenic cells (i.e., CSCs) [2], [41], [42]. However, here we show that previously identified CSC markers (e.g., CD34^+^CD38^−^ for AML, APL, and CML, CD44^+^CD24^−^ for breast cancer, CD133^+^ for glioblastoma and colon cancer, CD271^+^ for melanoma) do not necessarily filter out tumorigenic cells in these tumors, because both CSC^+^ and CSC^−^ cells can initiate tumor growth and give rise to both progenies (ie, CSC^+^ and CSC^−^ cells). Our data raise interesting questions regarding the plasticity of tumor hierarchy.

The CSC model posits that the initiation of tumor formation is driven by CSCs while the non-CSCs, which compose the bulk of cells in a tumor, have no or limited capacity for initiation of tumor formation [1], [35], [36]. On the other hand, CSCs were reported to be more quiescent than non-CSCs [41], [43]. If fundamental differences in the proliferative potential does exist between CSCs and non-CSCs, such differences should be easily detected by immunohistochemical or flow cytometric analysis of cell proliferation. However, here we showed that the proliferation and apoptosis of the putative CSCs (CSC^+^ cells) and non-CSCs (CSC^−^ cells) were remarkably similar in primary tumors and tumor cell lines, suggesting that the proliferative capacity of CSC^−^ cells might have been seriously underestimated in previous studies in which CSC^+^ and CSC^−^ cells were separately transplanted into the animals. Therefore, we established a cell tracing method designed to simulate the *in situ* environment where CSC^+^ and CSC^−^ cells coexist. In contrast to the previous reports that CSC^−^ cells had no or limited ability of proliferation and hence did not initiate tumor growth [35], [36], we showed that CSC^−^ cells exhibited similar proliferation or sustainability as CSC^+^ cells when both subsets were co-cultured *in vitro* or co-transplanted into the animals according to their original ratios. These data strongly suggest that, in addition to CSC^+^ cells, CSC^−^ cells also are capable of proliferation and tumorigenesis.

The CSC model depicts a tumor as a cellular hierarchy in which only CSCs lying at the apex have the ability of self-renewal and generating cells of the rest. Available evidence supporting the CSC model mostly comes from tumor-forming studies. As mentioned earlier, the tumor-forming assay may not be sufficient to demonstrate a hierarchal organization since the tumor-forming capacity of tumor cells could be dramatically influenced by the intrinsic and extrinsic factors [33], [34], especially when different subsets of tumor cells are studied separately in a foreign milieu. On the other hand, although some studies showed that CSC^−^ (CD133^−^) cells in glioblastoma [38] and colon cancer [39] could initiate tumor growth, whether or not there is a CSC marker based hierarchy in these tumors were not investigated extensively. In the present study, we investigated the fate of the cells derived from putative CSCs (CSC^+^ cells) and non-CSCs (CSC^−^ cells) by cell tracing under condition where the two subsets coexisted. We found both CSC^+^ and CSC^−^ cells could be detected in progenies derived from CSC^+^ (or CSC^−^) cells both *in vitro* and *in vivo*. Through serial single-cell derived subline construction with cells from tumor cell lines, we further demonstrated that CSC^−^ as well as CSC^+^ cells from CSC marker expressing tumors could invariably give rise to both progenies, and the proliferative or tumorigenic capacity of CSC^+^ (or CSC^−^) cells was maintained among different generations, regardless of the origins (CSC^+^-derived or CSC^−^-derived). These data provide strong evidences that there is no common CSC marker based hierarchy in these tumors.

Cell surface markers have been widely used to distinguish CSCs from non-CSCs and most of the previous studies suggested that CSCs were restricted to a rare population of tumor cells [35], [36]. However, our data suggest that the expression of cell surface markers is more complex than previously recognized. Consistent with some other reports [38], [39], we showed by flow cytometric analysis that the percentage of CSC marker positive cells varied enormously (ranging from 0.4% in an AML to as high as 82.7% in a colon cancer) in CSC marker expressing tumors and that a considerable number of primary tumors or tumor cell lines did not express those CSC markers. Through quantitative RT-PCR, we further revealed that cells (both CSC^+^ and CSC^−^) from CSC marker expressing tumors but not cells from CSC marker non-expressing tumors expressed CSC marker mRNA, suggesting that the CSC marker expressing and non-expressing tumors might originate from different types of tumor cells. Notably, in contrast to both the CSC model and clonal evolution model, in which the phenotypic heterogeneity is attributed to epigenetic and genetic changes, respectively, we show that the expression of CSC markers in cells from CSC marker expressing tumor cell lines can be dynamic. Such phenomenon was also noted by others in some human primary tumors [44]–[46] and tumor cell lines [45], [46]. Whether such phenomenon exists in other primary tumors warrants further investigation.

The present data show that both CSC^+^ and CSC^−^ cells have the ability of tumorigenesis and that the expression of CSC markers is reversible. However, we do not rule out functional heterogeneity between the two subsets as CSC^+^ cells exhibited higher tumorigenicity than CSC^−^ cells when they were transplanted separately into mice. Previously, CSC^+^ cells were reported to exhibit greater immune tolerance (e.g., CD271^+^ human melanoma cells [29]) and higher secretion of growth factors (e.g., CD133^+^ human glioblastoma cells [47]) than CSC^−^ cells. Hence, the higher tumorigenicity of CSC^+^ cells in xenotransplantation may either be due to their better adaptation to the foreign milieu and/or their ability to secrete growth factors that are critical for cell survival and proliferation. Whether the CSC^+^ and CSC^−^ cells may show overt differences in tumor initiation, metastasis, and resistance to chemotherapy or radiotherapy in the human milieu remains to be further investigated. A better understanding of the underlying mechanisms of the functional heterogeneity might provide important clues for the development and evaluation of novel anticancer therapies.

### Conclusions

The present investigation shows the limitation of using common CSC markers to identify a cell population (ie, CSCs) that are *exclusively* capable of proliferating and initiating tumor growth in the tumor cell lines we studied. Our data are more supportive of the clonal evolution model in which most of the tumor cells are capable of proliferation and tumorigenesis and the functional heterogeneity of tumor cells is attributable to random or stochastic influences (intrinsic or extrinsic). This conclusion is based on the findings that coexisting putative CSCs (CSC^+^ cells) and non-CSCs (CSC^−^ cells) exhibited similar capacity for proliferation and tumorigenesis and that both subsets could give rise to CSC^+^ and CSC^−^ progenies. Our results suggest the limitations of using these markers independently to differentiate cancer stem cells from non-tumorigenic cells due to the phenotypic plasticity of tumor cells.

## Materials and Methods

### Ethics statement

Human tumor specimens were obtained from patients after they signed a consent form written in Chinese, which according to a protocol approved by the Medical Ethics Committee of Changhai Hospital. All animal experiments were approved by the Institutional Animal Care and Use Committee of Changhai Hospital (protocol no. 2009-0071).

### Tumor cell preparation

Human tumor specimens were obtained from consenting patients according to a protocol approved by the Medical Ethics Committee of Changhai Hospital. Fresh leukemia (AML, APL, and CML) peripheral blood cells were enriched by Ficoll-density gradient centrifugation and washed in Iscove's modified Dulbecco's medium (IMDM) containing 5% fetal calf serum. Solid primary tumor samples (breast cancer, glioblastoma, colon cancer, and melanoma) or xenografts were mechanically dissociated and then digested in a trypsin-free (to maintain epitope integrity) medium containing 150 μg/mL Collagenase Type IV, 2 μg/mL DNase type I and 10 μg/mL hyaluronidase type V (Sigma) for 2 hr at 37°C. The resulting cell suspension was filtered through a 38-μm nylon mesh and single cells were harvested.

### Cell labeling

Cells from tumor cell lines were infected with 1 mL of EGFP or DsRed recombinant lentiviral supernatant containing 8 µg/mL polybrene (Invitrogen) with a multiplicity of infection of 1∶5 for 2 h at 37°C. Transgenic cells that stably expressed EGFP or DsRed were isolated to construct EGFP- (or DsRed-) labeled cell sublines.

### Flow cytometric analysis and cell sorting

After washing with PBS (100 mM, pH 7.2), cells were re-suspended in 500 μL Buffer1 (100 mM PBS containing 0.5% BSA). Unlabeled or EGFP-labeled cells from solid tumors were incubated for 1 hour at 4°C with PE-conjugated antibody specific to the CSC markers (CD133 for glioblastoma and colon cancer, CD271 for melanoma) or PE-conjugated mouse IgG1 isotype control antibody (Miltenyi), while DsRed-labeled cells were incubated with FITC-conjugated antibodies. Cells were washed twice, re-suspended in 500 μL Buffer1, and then analyzed or isolated on a MoFlo cell sorter (Dako, with the gate set on the basis of isotype control staining profiles). In the case of leukemia (AML, APL, and CML), unlabeled or GFP-labeled cells were double stained with PE-conjugated mouse anti-human CD34 (BD Biosciences) and APC-conjugated mouse anti-human CD38 antibody (BD Biosciences), while DeRed-labeled cells were double stained with FITC-conjugated mouse anti-human CD34 (BD Biosciences) and APC-conjugated mouse anti-human CD38 antibody. For breast cancer, unlabeled or GFP-labeled cells were double stained with PE-conjugated mouse anti-human CD44 (BD Biosciences) and APC-conjugated mouse anti-human CD24 antibody (BD Biosciences), while DeRed-labeled cells were double stained with FITC-conjugated mouse anti-human CD44 (BD Biosciences) and APC-conjugated mouse anti-human CD24 antibody. Then, different cell subsets were analyzed or isolated on a MoFlo cell sorter as described above. The purity of sorted cells was evaluated by flow cytometry. For all samples, sorted CSC^+^ subsets contained >96% of CSC^+^ cells, while sorted CSC^−^ subsets contained >99% of CSC^−^ cells.

### Co-culture of CSC^+^ and CSC^−^ tumor cells

DsRed-labeled CSC^+^ and EGFP-labeled CSC^−^ cells originating from the same tumor cell lines were mixed according to their original ratio. The mixed cells were cultured in 12-well plates (3×10^5^ cells per well) until confluent (approximately 1×10^6^), which was arbitrarily classified as passage 1 (P1). Then, cells were passaged using a 1∶3 dilution and grown to confluency (P2). We repeated this procedure until passage 20. The ratio of DsRed:EGFP cells in different passages was determined by flow cytometry.

### Transplantation of tumor cells

All animal experiments were approved by the Institutional Animal Care and Use Committee of Changhai Hospital. Different numbers (1×10^3^, 1×10^4^, and 1×10^5^) of CSC^+^ and CSC^−^ cells were suspended in a 1∶1 mixture of media and matrigel (BD Biosciences) and injected under the renal capsule of anesthetized NOD-SCID mice (8 weeks of age, 3 animals for each cell dose) that had been sublethally irradiated (350 centigray). In the case of co-transplantation, 1×10^5^ mixed cells (DsRed-labeled CSC^+^ and EGFP-labeled CSC^−^ cells were mixed to the original ratio) were injected as described above. Animals were sacrificed between 15 and 20 weeks post-transplantation.

### Single-cell derived subline construction

Single CSC^+^ or CSC^−^ cell from the CSC marker expressing tumor cell lines were seeded into the 96-well plates. Single-cell derived clones were cultured in the 12-well plates (one clone per well) until confluent (approximately 1×10^6^), which was arbitrarily classified as P1. Then, cells were passaged using a 1∶3 dilution and grown to confluency (P2). We repeated this procedure until passage 20.

### Statistical analysis

Data are presented as mean ± SEM. Statistical significance was tested using SPSS15.0 software, with *t*-tests for 2-group comparisons or analysis of variance (ANOVA) for multiple group comparisons. Tumorigenic difference between CSC^+^ and CSC^−^ cells from the same tumor cell lines was tested using Extreme Limiting Dilution Analysis software (available from the Bioinformatics section of the Walter and Eliza Hall Institute of Medical Research, http://bioinf.wehi.edu.au/software/elda/index.html) [48].

## Supporting Information

Figure S1 **The expression of CSC marker mRNA in primary tumors.** The CSC marker mRNA expression in primary tumors was detected by RT-PCR. Photographs show the RT-PCR products of AML, APL, CML, breast cancer, glioblastoma, colon cancer, and melanoma samples, respectively. Note that some samples (two samples in glioblastoma, two samples in colon cancer, and one sample in melanoma) do not express CSC marker mRNA. Related to Figure 1.
(JPG)

Figure S2 **CSC^+^ and CSC^−^ tumor cells can generate both progenies.** The percentage of CSC marker positive cells in the original tumor cell lines or SCDSLs (passage 1 and passage 20) of THP-1 (A), HL60 (B), K562 (C), MDA-MB-231(D), U251 (E), Caco-2 (F), SW480 (G) and SW620 (H) was analyzed by flow cytometry. Box plots (left) show the percentage of CSC marker positive cells in SCDSLs, with the whiskers representing the minimum and maximum values, the central lines representing the median value, and the boxes representing the 25th and 75th percentile. Histograms (right) show the percentage of CSC marker positive cells in original tumor cell lines and SCDSLs. Data of SCDSLs represent mean ± SEM from 100 samples; Data of original tumor cell lines represent mean ± SEM from 3 independent experiments; ** *P*<0.01 by independent *t*-test. Related to Figure 4.
(JPG)

Figure S3 **Clone forming efficiency of the CSC^+^ and CSC^−^ cells of leukemia** (**A**)**, breast cancer** (**B**)**, glioblastoma** (**C**)**, colon cancer** (**D**)**, and melanoma** (**E**) **cells lines.** Notably, the clone forming efficiency of CSC^−^ cells in each and every tumor cell line is far much higher than the percentage of CSC^+^ cells (false negative cells, usually lower than 0.1%) existed in the CSC-cells sorted by FACS. Data represent mean ± SEM from 3 independent experiments; ** *P*<0.01 by independent *t*-test. Related to Figure 4.
(JPG)

Figure S4 **Variability in CSC marker based hierarchy in CSC marker expressing tumors.** The percentage of CSC marker positive cells in SCDSLs from THP-1, HL60, K562, MDA-MB-231, U251, HT-29, SW480 and SW620 was analyzed by flow cytometry when the cell quantity reached approximately 1×10^6^. Box plots show the percentage of CSC marker positive cells in single CSC^+^-derived (A) and CSC^−^-derived (B) sublines from different generations, with the whiskers representing the minimum and maximum values, the central lines representing the median value, and the boxes representing the 25th and 75th percentile. Histograms show the percentage of CSC marker positive cells in single CSC^+^-derived (C) and CSC^−^-derived (D) sublines from different generations. Data represent mean ± SEM from 100 samples, each from one independent serial SCDSL construction. Related to Figure 5.
(JPG)

Figure S5 **Proliferation and tumorigenesis of the CSC^+^** (**or CSC^−^**) **cells from different generations are comparable.** Histograms show the percentage of Ki-67 positive cells, apoptotic index, and the frequency of tumorigenic cells of CSC^+^ (A) and CSC^−^ (B) from different generations of THP-1, HL60, K562, MDA-MB-231, U251, HT-29, SW480 and SW620. Data of the proliferative and apoptotic indices represent mean ± SEM from 3 independent experiments. Data of the frequency of tumorigenic cells were calculated by extreme limiting dilution analysis software. Related to Figure 6.
(JPG)

Table S1 Tumorigenesis analysis of separately transplanted CSC^+^ and CSC^−^ tumor cells. For each tumor cell line, different doses (1×10^3^, 1×10^4^, or 1×10^5^ cells) of CSC^+^ (or CSC^−^) cells were transplanted into the animals (n = 3 for each cell dose), respectively. Extreme Limiting Dilution Analysis software was used to estimate the frequency of tumorigenic cells. The frequency of tumorigenic cells was compared between CSC^+^ or CSC^−^ cells from the same tumor cell line, * *P*<0.5, ** *P*<0.01.
(DOC)

Table S2 CSC^+^ and CSC^−^ cells are capable of generating both progenies. In *in vitro* study, DsRed-labeled CSC^+^ and EGFP-labeled CSC^−^ cells originating from the same tumor cell lines were mixed according to their original ratios and co-cultured in serum-containing medium for 20 passages; In *in vivo* study, DsRed-labeled CSC^+^ and EGFP-labeled CSC^−^ cells originating from the same tumor cell lines were mixed according to their original ratios and co-transplanteded into the animals to generate xenografts (see Experimental Procedures for details). Then, the percent of CSC-positive cells in CSC^+^-(DsRed-labeled) and CSC^−^-derived (EGFP-labeled) population was analyzed by flow cytometry.
(DOC)

Methods S1 **Supplemental experimental methods.**
(DOC)
