# Supplementary figures and images for: Tumor Cells Positive and Negative for the Common Cancer Stem Cell Markers Are Capable of Initiating Tumor Growth and Generating Both Progenies
Source: PLoS One. 2013 Jan 21;8(1):e54579. doi: 10.1371/journal.pone.0054579 (PMC3549952; doi:10.1371/journal.pone.0054579)

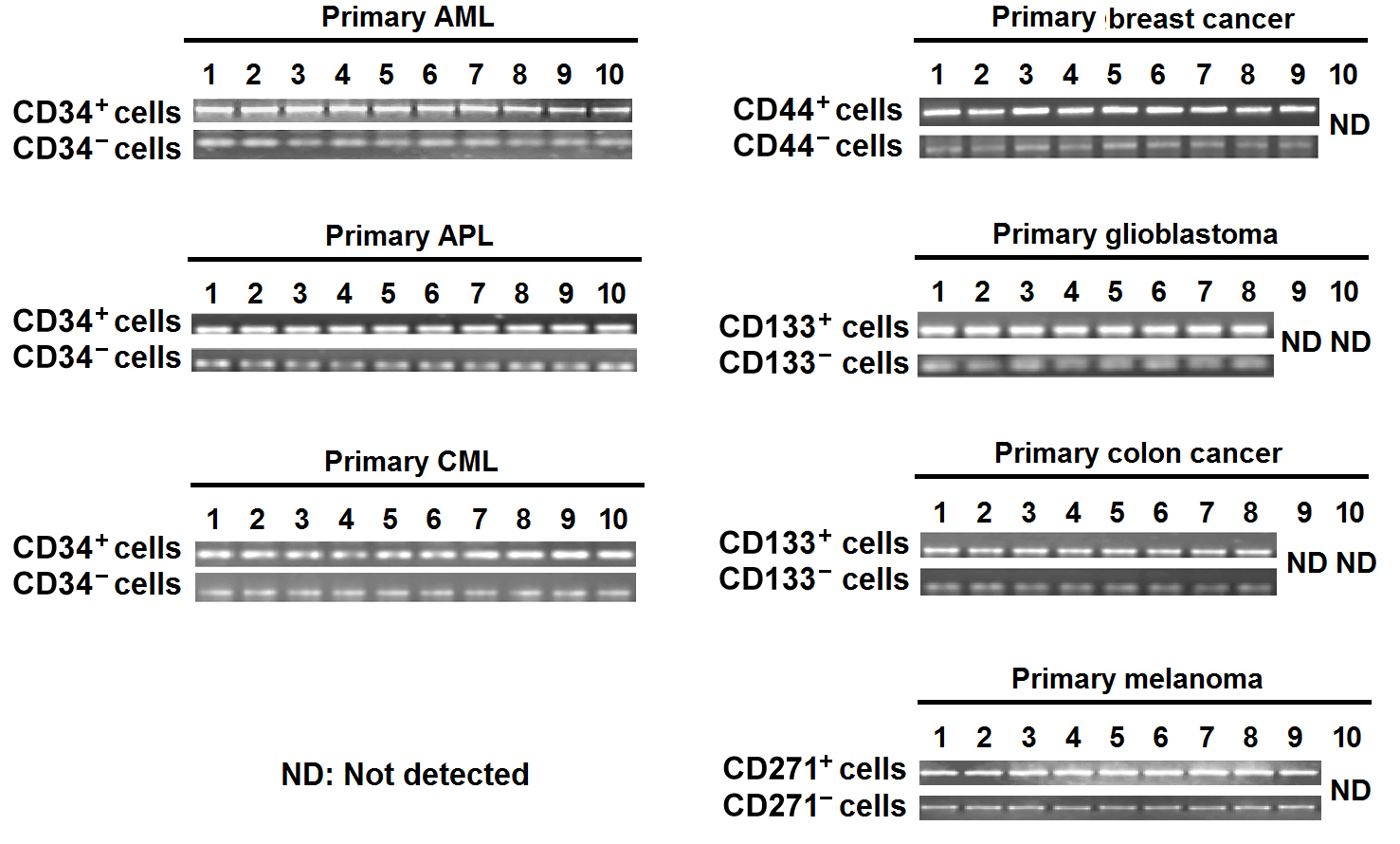

Supplement: Figure S1 — The expression of CSC marker mRNA in primary tumors. The CSC marker mRNA expression in primary tumors was detected by RT-PCR. Photographs show the RT-PCR products of AML, APL, CML, breast cancer, glioblastoma, colon cancer, and melanoma samples, respectively. Note that some samples (two samples in glioblastoma, two samples in colon cancer, and one sample in melanoma) do not express CSC marker mRNA. Related to Figure 1. (JPG) [file pone.0054579.s001.jpg]

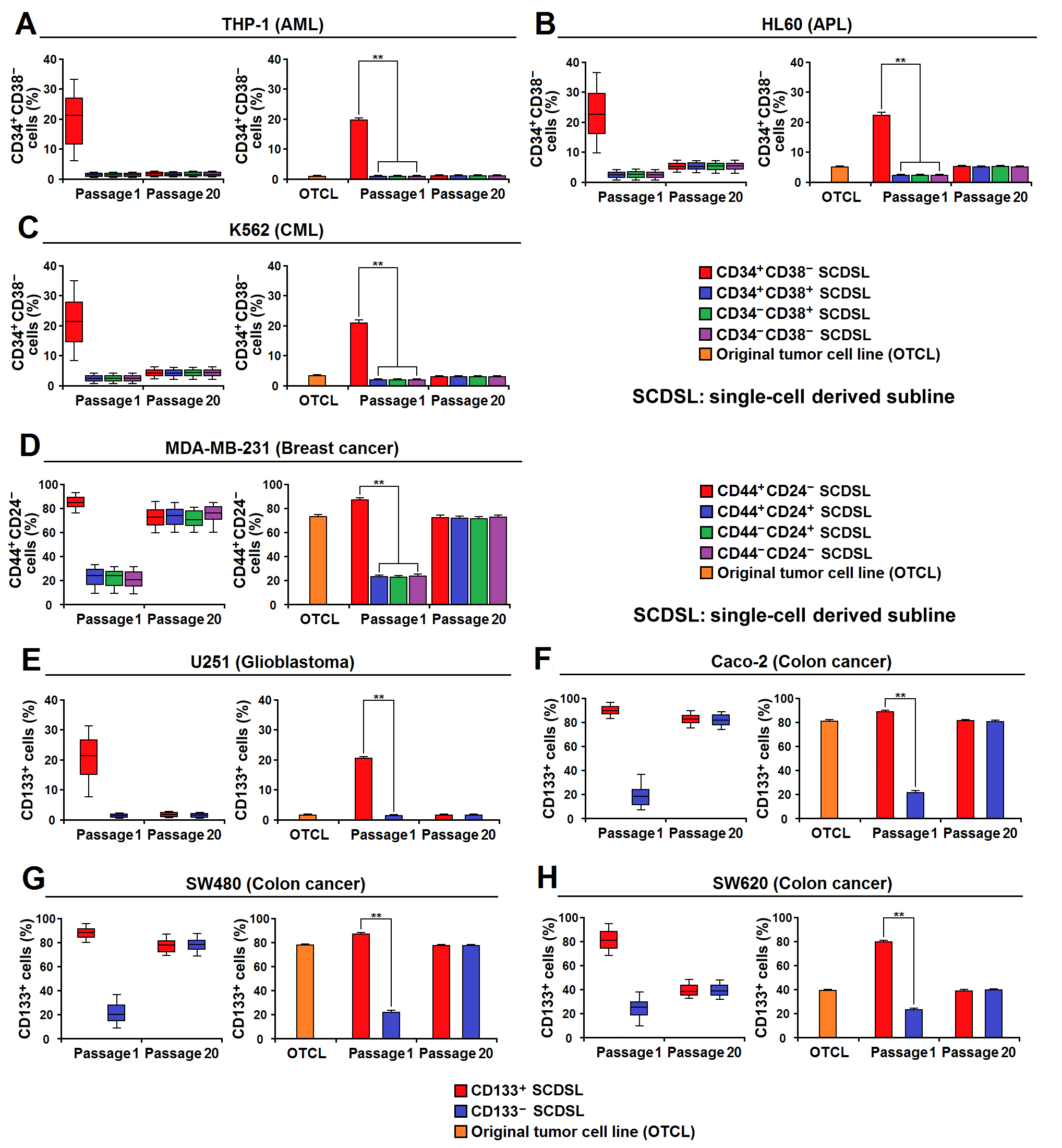

Supplement: Figure S2 — CSC+ and CSC− tumor cells can generate both progenies. The percentage of CSC marker positive cells in the original tumor cell lines or SCDSLs (passage 1 and passage 20) of THP-1 (A), HL60 (B), K562 (C), MDA-MB-231(D), U251 (E), Caco-2 (F), SW480 (G) and SW620 (H) was analyzed by flow cytometry. Box plots (left) show the percentage of CSC marker positive cells in SCDSLs, with the whiskers representing the minimum and maximum values, the central lines representing the median value, and the boxes representing the 25th and 75th percentile. Histograms (right) show the percentage of CSC marker positive cells in original tumor cell lines and SCDSLs. Data of SCDSLs represent mean ± SEM from 100 samples; Data of original tumor cell lines represent mean ± SEM from 3 independent experiments; ** P<0.01 by independent t-test. Related to Figure 4. (JPG) [file pone.0054579.s002.jpg]

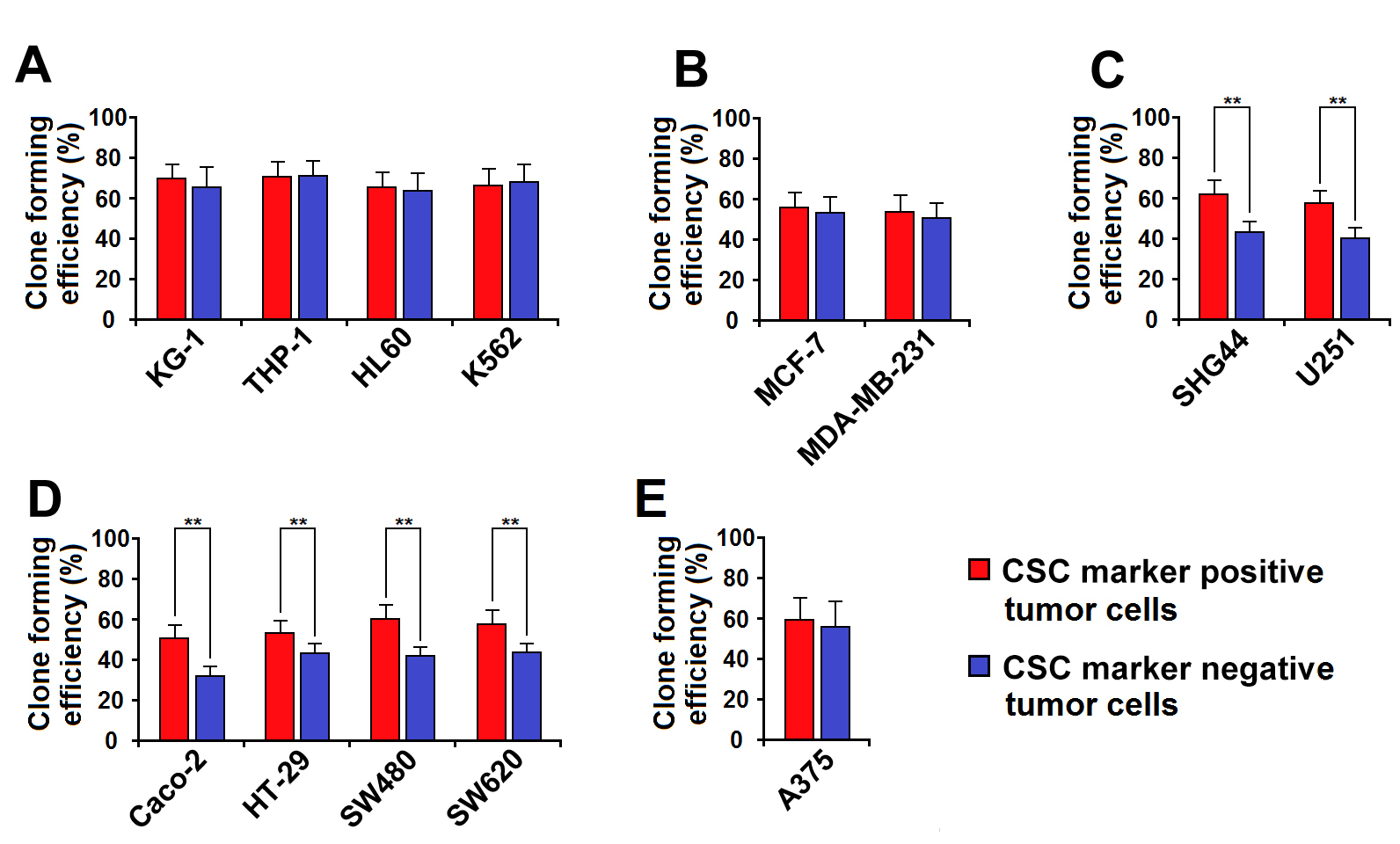

Supplement: Figure S3 — Clone forming efficiency of the CSC+ and CSC− cells of leukemia (A), breast cancer (B), glioblastoma (C), colon cancer (D), and melanoma (E) cells lines. Notably, the clone forming efficiency of CSC− cells in each and every tumor cell line is far much higher than the percentage of CSC+ cells (false negative cells, usually lower than 0.1%) existed in the CSC-cells sorted by FACS. Data represent mean ± SEM from 3 independent experiments; ** P<0.01 by independent t-test. Related to Figure 4. (JPG) [file pone.0054579.s003.jpg]

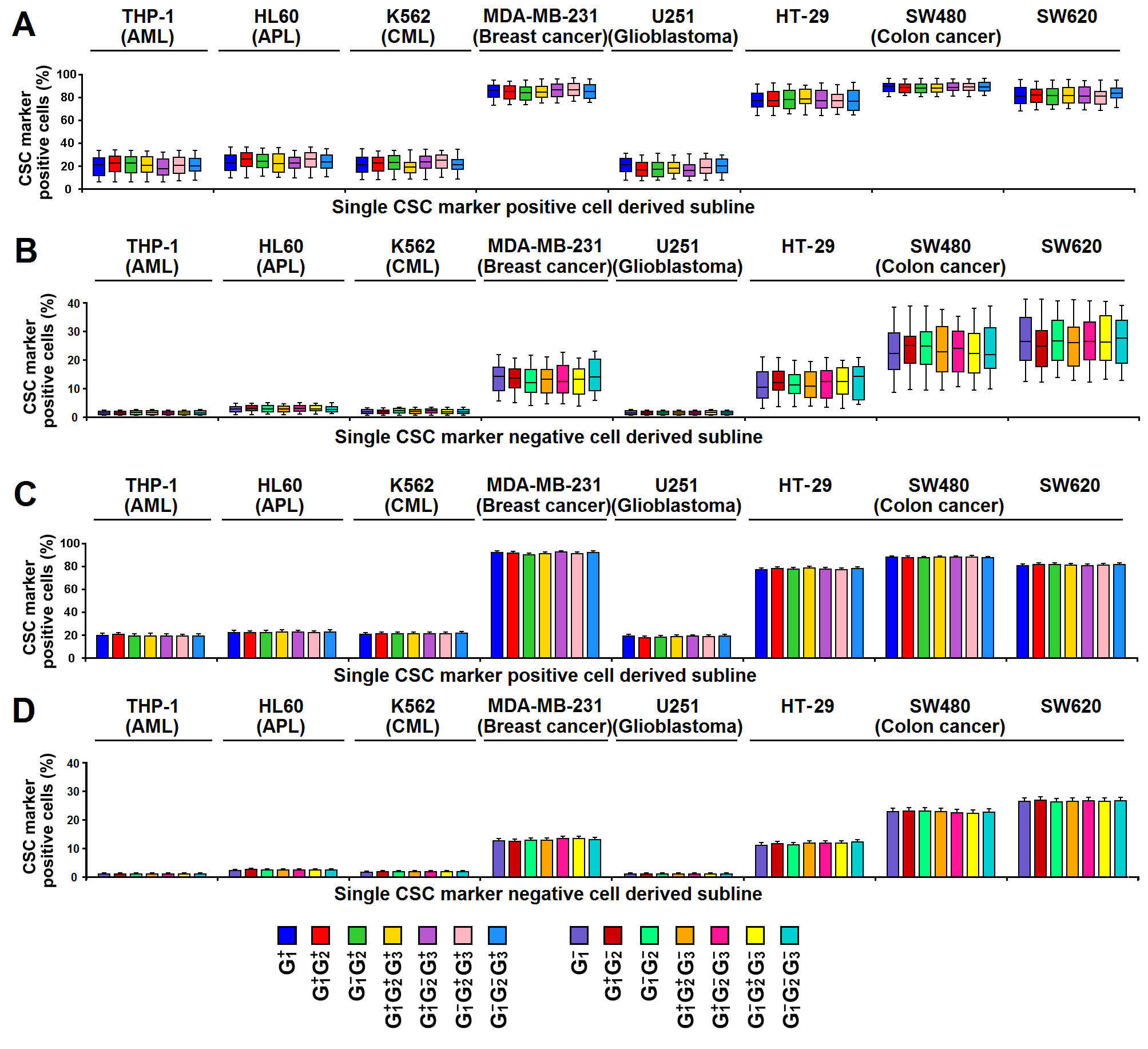

Supplement: Figure S4 — Variability in CSC marker based hierarchy in CSC marker expressing tumors. The percentage of CSC marker positive cells in SCDSLs from THP-1, HL60, K562, MDA-MB-231, U251, HT-29, SW480 and SW620 was analyzed by flow cytometry when the cell quantity reached approximately 1×10^6^. Box plots show the percentage of CSC marker positive cells in single CSC+-derived (A) and CSC−-derived (B) sublines from different generations, with the whiskers representing the minimum and maximum values, the central lines representing the median value, and the boxes representing the 25th and 75th percentile. Histograms show the percentage of CSC marker positive cells in single CSC+-derived (C) and CSC−-derived (D) sublines from different generations. Data represent mean ± SEM from 100 samples, each from one independent serial SCDSL construction. Related to Figure 5. (JPG) [file pone.0054579.s004.jpg]

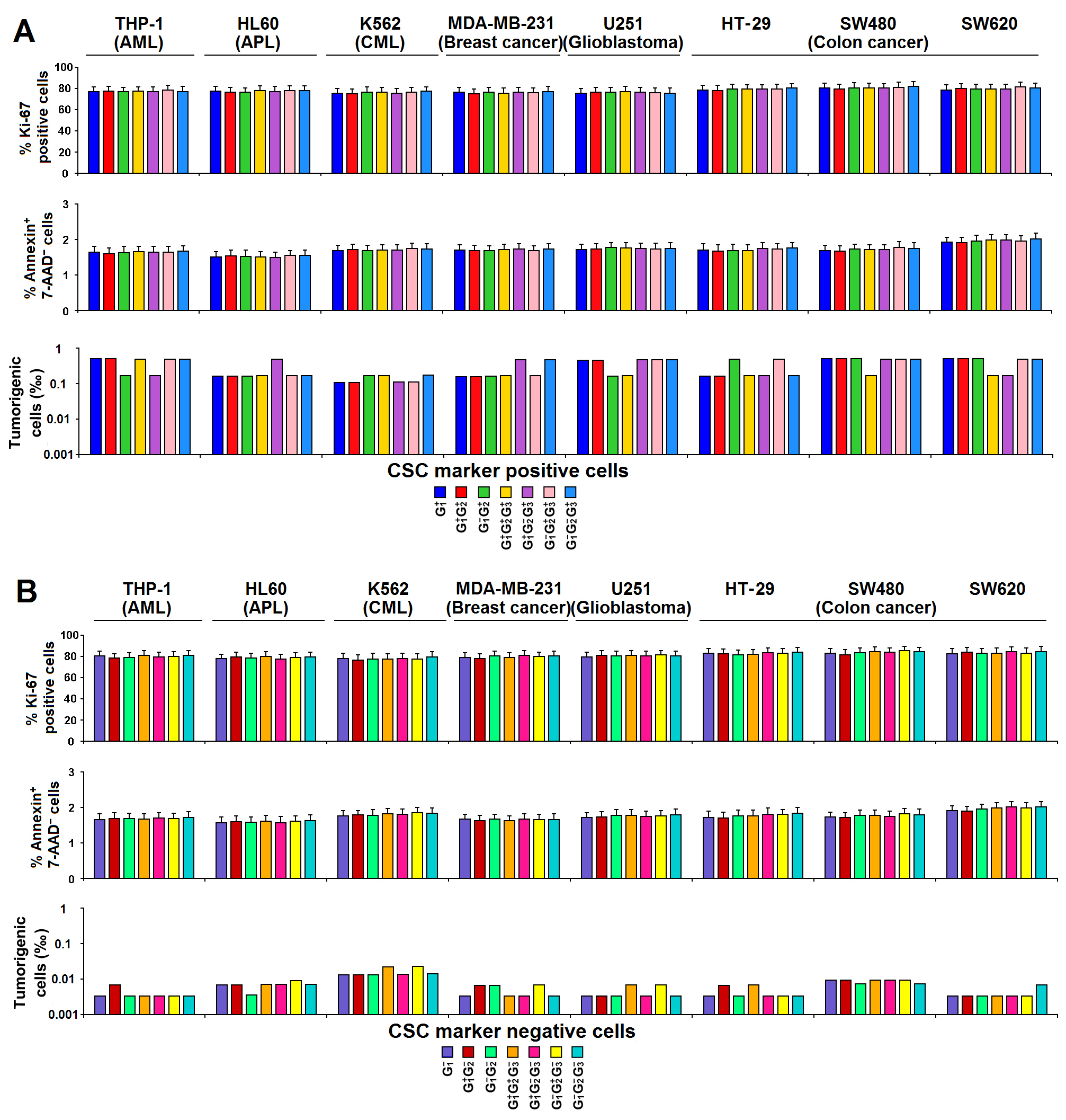

Supplement: Figure S5 — Proliferation and tumorigenesis of the CSC+ (or CSC−) cells from different generations are comparable. Histograms show the percentage of Ki-67 positive cells, apoptotic index, and the frequency of tumorigenic cells of CSC+ (A) and CSC− (B) from different generations of THP-1, HL60, K562, MDA-MB-231, U251, HT-29, SW480 and SW620. Data of the proliferative and apoptotic indices represent mean ± SEM from 3 independent experiments. Data of the frequency of tumorigenic cells were calculated by extreme limiting dilution analysis software. Related to Figure 6. (JPG) [file pone.0054579.s005.jpg]
