# Supplementary material for: Tumor Cells Positive and Negative for the Common Cancer Stem Cell Markers Are Capable of Initiating Tumor Growth and Generating Both Progenies
Source: PLoS One. 2013 Jan 21;8(1):e54579. doi: 10.1371/journal.pone.0054579 (PMC3549952; doi:10.1371/journal.pone.0054579)
Supplement: Table S1 — Tumorigenesis analysis of separately transplanted CSC+ and CSC− tumor cells. For each tumor cell line, different doses (1×10^3^, 1×10^4^, or 1×10^5^ cells) of CSC+ (or CSC−) cells were transplanted into the animals (n = 3 for each cell dose), respectively. Extreme Limiting Dilution Analysis software was used to estimate the frequency of tumorigenic cells. The frequency of tumorigenic cells was compared between CSC+ or CSC− cells from the same tumor cell line, * P<0.5, ** P<0.01. (DOC) [file pone.0054579.s006.doc]

~~Table S1. Tumorigenesis analysis of separately transplanted CSC~~^~~+~~^ ~~and CSC~~^~~-~~^ ~~tumor cells.~~

| Cell line | Cell  subset | Xenografts/Injections | | | Frequency of tumorigenic cells |
| --- | --- | --- | --- | --- | --- |
|  |  | 1×10^3^ cells | 1×10^4^ cells | 1×10^5^ cells |  |
| KG-1 | CSC^+^ | 1/3 | 3/3 | 3/3 | 1/2164 |
|  | CSC^-^ | 0/3 | 1/3 | 2/3 | 1/68078** |
| THP-1 | CSC^+^ | 1/3 | 3/3 | 3/3 | 1/2164 |
|  | CSC^-^ | 0/3 | 0/3 | 1/3 | 1/280030** |
| HL60 | CSC^+^ | 0/3 | 3/3 | 3/3 | 1/4170 |
|  | CSC^-^ | 0/3 | 1/3 | 2/3 | 1/68078** |
| K562 | CSC^+^ | 0/3 | 2/3 | 3/3 | 1/10720 |
|  | CSC^-^ | 0/3 | 1/3 | 1/3 | 1/135931** |
| MCF-7 | CSC^+^ | 1/3 | 3/3 | 3/3 | 1/2164 |
|  | CSC^-^ | 0/3 | 0/3 | 1/3 | 1/280030** |
| MDA-  MB-231 | CSC^+^ | 0/3 | 3/3 | 3/3 | 1/4170 |
|  | CSC^-^ | 0/3 | 0/3 | 2/3 | 1/108957** |
| SHG44 | CSC^+^ | 1/3 | 3/3 | 3/3 | 1/2164 |
|  | CSC^-^ | 0/3 | 1/3 | 1/3 | 1/135931** |
| U251 | CSC^+^ | 1/3 | 3/3 | 3/3 | 1/2164 |
|  | CSC^-^ | 0/3 | 0/3 | 1/3 | 1/280030** |
| Caco-2 | CSC^+^ | 1/3 | 3/3 | 3/3 | 1/2164 |
|  | CSC^-^ | 0/3 | 1/3 | 1/3 | 1/135931** |
| HT-29 | CSC^+^ | 0/3 | 3/3 | 3/3 | 1/4170 |
|  | CSC^-^ | 0/3 | 0/3 | 1/3 | 1/280030** |
| SW480 | CSC^+^ | 1/3 | 3/3 | 3/3 | 1/2164 |
|  | CSC^-^ | 0/3 | 0/3 | 2/3 | 1/108957** |
| SW620 | CSC^+^ | 1/3 | 3/3 | 3/3 | 1/2164 |
|  | CSC^-^ | 0/3 | 0/3 | 1/3 | 1/280030** |
| A375 | CSC^+^ | 0/3 | 3/3 | 3/3 | 1/4170 |
|  | CSC^-^ | 0/3 | 0/3 | 3/3 | 1/43259* |
