# Supplementary material for: Tumor Cells Positive and Negative for the Common Cancer Stem Cell Markers Are Capable of Initiating Tumor Growth and Generating Both Progenies
Source: PLoS One. 2013 Jan 21;8(1):e54579. doi: 10.1371/journal.pone.0054579 (PMC3549952; doi:10.1371/journal.pone.0054579)
Supplement: Table S2 — CSC+ and CSC− cells are capable of generating both progenies. In in vitro study, DsRed-labeled CSC+ and EGFP-labeled CSC− cells originating from the same tumor cell lines were mixed according to their original ratios and co-cultured in serum-containing medium for 20 passages; In in vivo study, DsRed-labeled CSC+ and EGFP-labeled CSC− cells originating from the same tumor cell lines were mixed according to their original ratios and co-transplanteded into the animals to generate xenografts (see Experimental Procedures for details). Then, the percent of CSC-positive cells in CSC+-(DsRed-labeled) and CSC−-derived (EGFP-labeled) population was analyzed by flow cytometry. (DOC) [file pone.0054579.s007.doc]

Table S2. CSC^+^ and CSC^-^ cells are capable of generating both progenies

| Cell line | Percent of CSC-positive cells  in CSC^+^-derived population | | Percent of CSC-positive cells  in CSC^-^-derived population | |
| --- | --- | --- | --- | --- |
|  | *in vitro*  (%, passage 20) | *in vivo*  (%, passage 3) | *in vitro*  (%, passage 20) | *in vivo*  (%, passage 3) |
| KG-1 | 21.5 ± 1.6 | 21.7 ± 1.5 | 22.7 ± 1.4 | 21.9 ± 1.1 |
| THP-1 | 1.8 ± 0.3 | 1.7 ± 0.2 | 1.9 ± 0.3 | 1.6 ± 0.2 |
| HL60 | 5.2 ± 0.7 | 4.8 ± 0.6 | 5.0 ± 0.6 | 4.3 ± 0.5 |
| K562 | 3.5 ± 0.4 | 3.6 ± 0.4 | 3.7 ± 0.5 | 3.4 ± 0.4 |
| MCF-7 | 2.7 ± 0.4 | 2.3± 0.5 | 2.6 ± 0.6 | 2.1± 0.5 |
| MDA- MB-231 | 68.6 ±8.0 | 73.2± 7.5 | 72.3 ±7.6 | 69.7± 7.3 |
| SHG44 | 1.8 ± 0.2 | 1.8 ± 0.2 | 1.7 ± 0.2 | 1.8 ± 0.2 |
| U251 | 1.7 ± 0.2 | 1.9 ± 0.2 | 1.9 ± 0.2 | 1.8 ± 0.2 |
| Caco-2 | 83.6 ± 5.2 | 80.4 ± 7.1 | 81.2 ± 3.7 | 79.3 ± 5.6 |
| HT-29 | 29.8 ± 1.9 | 31.7 ± 2.2 | 31.2 ± 1.6 | 30.9 ± 2.0 |
| SW480 | 76.5 ± 5.8 | 79.3 ± 7.5 | 79.7 ± 4.3 | 72.5 ± 8.9 |
| SW620 | 39.4 ± 3.1 | 39.7 ± 2.8 | 37.6 ± 2.5 | 42.0 ± 3.7 |
| A375 | 14.4 ± 0.9 | 14.6 ± 1.1 | 14.9 ± 0.8 | 15.4 ± 1.2 |
