# Supplementary material for: Tumor Cells Positive and Negative for the Common Cancer Stem Cell Markers Are Capable of Initiating Tumor Growth and Generating Both Progenies
Source: PLoS One. 2013 Jan 21;8(1):e54579. doi: 10.1371/journal.pone.0054579 (PMC3549952; doi:10.1371/journal.pone.0054579)
Supplement: Methods S1 — Supplemental experimental methods. (DOC) [file pone.0054579.s008.doc]

**Supplemental experimental methods**

***Tumor cell lines and animals.*** Human AML (KG-1 and THP-1), APL (HL60), CML (K562), breast cancer (MCF-7 and MDA-MB-231), glioblastoma (SHG44, U251, U37, and U81), colon cancer (Caco-2, COLO320, HT-29, LoVo, LS174T, SW480, and SW620), and melanoma (A375) cell lines were purchased from the Shanghai Institute of Biochemistry and Cell Biology (Shanghai, China). Cells were maintained in RPMI1640 (Invitrogen) containing 10% fetal bovine serum (Invitrogen), 100 U/mL penicillin, and 100 μg/mL streptomycin, within a humidified atmosphere containing 5% CO_2_ at 37°C.

NOD/SCID mice were purchased from Shanghai SLAC Laboratory Animal Co. Ltd and maintained in the Laboratory Animal Research Center of Changhai Hospital. All animal experiments were approved by the Institutional Animal Care and Use Committee of Changhai Hospital.

***Quantitative reverse transcriptase PCR.*** Total RNA was extracted from 1×10^5^ cells using the RNeasy RNA Mini Kit (Qiagen). First strand cDNA was synthesized using PowerScipt reverse transcriptase (Clontech). The following gene-specific primer pairs were used for quantitative PCR:

*CD34*: Forward, 5'-CTTTCAACCACTAGCACTAGCC-3';

Reverse, 5'-TGCCCTGAGTCAATTTCACTTC-3'

*CD44*: Forward, 5'- CCCATCCCAGACGAAGACAG-3';

Reverse, 5'- ACCATGAAAACCAATCCCAGG-3'

*CD133*: Forward, 5'-TGGGGCTGCTGTTTATTATTCT-3';

Reverse, 5'-TGCCACAAAACCATAGAAGATG-3'

*CD271*: Forward, 5'-TACATAGCCTTCAAGAGGTGG-3';

Reverse, 5'-GTTGAGAAGCTTCTCCACCTC-3'

*GAPDH*: Forward, 5'- GCTGAGTATGTCGTGGAGTC-3';

Reverse, 5'- AGTTGGTGGTGCAGGATGC-3'

PCR was performed using a Fast Start Master SYBR Green Kit (Roche) on a LightCycler (Roche). Analysis was carried out using RealQuant software (Roche). The mRNA expression level of *CD34*, *CD133,* and *CD271* was normalized to that of *GAPDH*.

***Cell cycle assay.*** Single cell suspensions of solid tumor samples were incubated with FITC-conjugated antibodies specific to CSC markers (CD133 for glioblastoma and colon cancer, CD271 for melanoma) for 1 hour at 4°C, and then fixed in 70% ethanol overnight at -20°C. After treatment with RNaseA (50 μg/mL, Sigma), the cells were stained with propidium iodide (25 μg/mL, Sigma) for 30 min at 37°C, and then subjected to flow cytometric analysis. The proliferative index was calculated as the percentage of cells in S/G2/M-phase. In the case of leukemia (AML, APL, and CML), cells were stained with FITC-conjugated mouse anti-human CD34 and APC-conjugated mouse anti-human CD38 antibody for 1 hour at 4°C, and then stained with propidium iodide and analyzed as described above. In the case of breast cancer, cells were stained with FITC-conjugated mouse anti-human CD44 and APC-conjugated mouse anti-human CD24 antibody for 1 hour at 4°C, and then stained with propidium iodide and analyzed as described above.

***Ki-67 expression assay.*** Single cell suspensions of solid tumor samples were fixed in 70% ethanol overnight at -20°C. Fixed cells were incubated with FITC-conjugated antibodies specific to CSC markers (CD133 for glioblastoma and colon cancer, CD271 for melanoma) for 1 hour at 4°C, and then stained with PE-conjugated mouse anti-human Ki-67 for 20 min at room temperature. The samples were subsequently subjected to flow cytometric analysis. In the case of leukemia (AML, APL, and CML), cells were stained with FITC-conjugated mouse anti-human CD34 and APC-conjugated mouse anti-human CD38 antibody for 1 hour at 4°C, and then stained with PE-conjugated mouse anti-human Ki-67 and analyzed as described above. In the case of breast cancer, cells were stained with FITC-conjugated mouse anti-human CD44 and APC-conjugated mouse anti-human CD24 antibody for 1 hour at 4°C, and then stained with PE-conjugated mouse anti-human Ki-67 and analyzed as described above.

***Apoptosis assay.*** Single cell suspensions of solid tumor samples were incubated with PE-conjugated antibodies specific to CSC markers (CD133 for glioblastoma and colon cancer, CD271 for melanoma) for 1 hour at 4°C, and then stained with Annexin V-FITC (Beckman) and 7-Amino actinomycin D (7-AAD, Sigma) for 20 min at room temperature. The samples were subsequently subjected to flow cytometric analysis. Viable cells were unstained by Annexin V or 7-AAD, early apoptotic cells were stained by Annexin V but not 7-AAD, and late apoptotic cells were stained by Annexin V and 7-AAD. The apoptotic index was calculated as the percentage of Annexin V^+^ 7-AAD^-^ cells. In the case of leukemia (AML, APL and CML), cells were stained with PE-conjugated mouse anti-human CD34 and APC-conjugated mouse anti-human CD38 antibody for 1 hour at 4°C, and then stained with Annexin V-FITC/7-AAD and analyzed as described above. In the case of breast cancer, cells were stained with PE-conjugated mouse anti-human CD44 and APC-conjugated mouse anti-human CD24 antibody for 1 hour at 4°C, and then stained with Annexin V-FITC/7-AAD and analyzed as described above.
